# Derivation of embryonic stem cells from wild-derived mouse strains by nuclear transfer using peripheral blood cells

**DOI:** 10.1038/s41598-023-38341-0

**Published:** 2023-07-10

**Authors:** Naomi Watanabe, Michiko Hirose, Ayumi Hasegawa, Keiji Mochida, Atsuo Ogura, Kimiko Inoue

**Affiliations:** 1 RIKEN BioResource Research Center, Tsukuba, Ibaraki Japan; 2 Graduate School of Science and Technology, University of Tsukuba, Tsukuba, Ibaraki Japan

**Keywords:** Reprogramming, Embryonic stem cells

## Abstract

Wild-derived mouse strains have been extensively used in biomedical research because of the high level of inter-strain polymorphisms and phenotypic variations. However, they often show poor reproductive performance and are difficult to maintain by conventional in vitro fertilization and embryo transfer. In this study, we examined the technical feasibility of derivation of nuclear transfer embryonic stem cells (ntESCs) from wild-derived mouse strains for their safe genetic preservation. We used leukocytes collected from peripheral blood as nuclear donors without sacrificing them. We successfully established 24 ntESC lines from two wild-derived strains of CAST/Ei and CASP/1Nga (11 and 13 lines, respectively), both belonging to *Mus musculus castaneus*, a subspecies of laboratory mouse. Most (23/24) of these lines had normal karyotype, and all lines examined showed teratoma formation ability (4 lines) and pluripotent marker gene expression (8 lines). Two male lines examined (one from each strain) were proven to be competent to produce chimeric mice following injection into host embryos. By natural mating of these chimeric mice, the CAST/Ei male line was confirmed to have germline transmission ability. Our results demonstrate that inter-subspecific ntESCs derived from peripheral leukocytes could provide an alternative strategy for preserving invaluable genetic resources of wild-derived mouse strains.

## Introduction

The laboratory mouse (*Mus musculus domesticus*) is the most widely used laboratory animal species. It has the great advantages of being small, easy to breed, domesticated to human handling, and with a short generation turnover, usually within three months. Mice have provided experimental models in many research fields since the early 1900s, and more than 400 inbred strains have been established during the past century^1^. In addition, highly reproducible gene-manipulation systems, including transgenic or gene knockout/knockin techniques were established in the 1980s^2^. More recently, the establishment of ground state (naïve) embryonic stem cells (ESCs) with high pluripotent ability has greatly promoted genetics as well as stem cell researchers using mice, especially the standard strain C57BL/6^3^. Importantly, abundant mouse genetic information has been available because of whole genome sequencing technology developed in the 1990s following the human genome project^4^. The high reproductive performance of these mice and the applicability of basic assisted reproductive technologies (ART) such as superovulation and in vitro fertilization (IVF) enable safe cryopreservation and restoration of valuable mouse strains. All these advantages have made the laboratory mouse the most broadly used model animal for a diversity of basic research including immunology, cancer biology, and genetic diseases of humans. On the other hand, such selective breeding based on experimental utility has resulted in a low genetic diversity among laboratory mice that limits the number of polymorphisms available on their genome and phenotypic variability^5^.

To overcome these drawbacks in laboratory mice, recently captured wild mice (wild-derived mice) have been used in many laboratories. These wild-derived mouse strains are now available as inbred strains, ensuring reproducibility of experiments (http://www.informatics.jax.org/ and https://mus.brc.riken.jp/en/). They include different subspecies (*M. m. musculus*, *molossinus*, and *castaneus*) that carry a wide range of genetic polymorphisms and can produce fertile F1 offspring by natural mating with laboratory mice. It is estimated that the common ancestor of subspecies of wild-derived mice divided into these major subspecies one million years ago and spread throughout the Eurasian and African continents^6,7^. Thus, the wild-derived subspecies currently available in laboratories originated from broad areas of continents such as Europe, Russia, China, Southeast Asia, or Japan^8^. Therefore, they have high genetic diversity, contributing to many biomedical research areas using mice, based on their polymorphisms and physiological variations. A typical example is a Japanese wild-derived strain, MSM/Ms (*M. M. molossinus*), which shows unique characteristics such as smaller body size, resistance to high-fat-diet-induced diabetes, high locomotive activity, and resistance to age-onset tumorigenesis^9^. However, wild-derived strains generally have disadvantages including low reproductive performance and difficulty in applying basic reproductive technologies, such as IVF and embryo transfer^10^. Therefore, it is often difficult to collect enough numbers of embryos for cryopreservation of wild-derived strains, resulting in their maintenance as live mice in most laboratories and mouse repository centers. These technical difficulties necessities another genetic backup system to avoid the loss of their invaluable genetic resources.

ESCs are pluripotent stem cells that have ability to give rise to three primary germ layers but not extraembryonic tissues. Due to the recent technical advancements in mouse ESC derivation and maintenance using differentiation-cascade inhibitors^3,11^, the quality of mouse ESCs has greatly improved in conventional strains as well as more specialized strains such as the Non-Obese Diabetic (NOD) mouse strain^12,13^. As a result, maintenance of mouse strains in the form of ESCs is now technically feasible. However, reports on the derivation of ESCs from wild-derived mouse strains are still limited^14^, mainly due to the difficulty in preparing a sufficient number of high-quality blastocysts for ESC derivation.

At present, fertilization-derived embryos are not the sole source for derivation of ESCs. Somatic cell nuclear transfer (SCNT), a technique to produce cloned individuals from differentiated somatic cells, also makes it possible to reconstruct embryos that may be used to establish nuclear transfer ESCs (ntESCs). It has already been demonstrated that ntESCs are identical to fertilized embryo-derived ESCs in their quality as pluripotent stem cells, such as restoration of mice through chimeric mouse production^15–17^. Therefore, derivation of ntESCs from wild-derived mice is expected to be an alternative strategy for their genetic preservation and restoration of the strains, if necessary.

In this study, we aimed to determine whether ntESCs could be established from wild-derived mouse strains, which exhibit low reproductive activity, by inter-subspecies SCNT. We used peripheral blood cells (leukocytes) as nuclear donors that can be collected less invasively. We have previously confirmed that the genome of peripheral leukocytes can be fully reprogrammed to produce cloned mice^18^. Here, we show successful establishment of inter-subspecies ntESC lines from wild-derived strains, giving rise to offspring through germline transmission. Our results indicate that generation of inter-subspecies ntESCs provides an alternative strategy for preserving invaluable genetic resources of wild-derived mouse strains.

## Results

### Establishment of ntESCs using leukocytes from wild-derived strains

**Figure 1:**
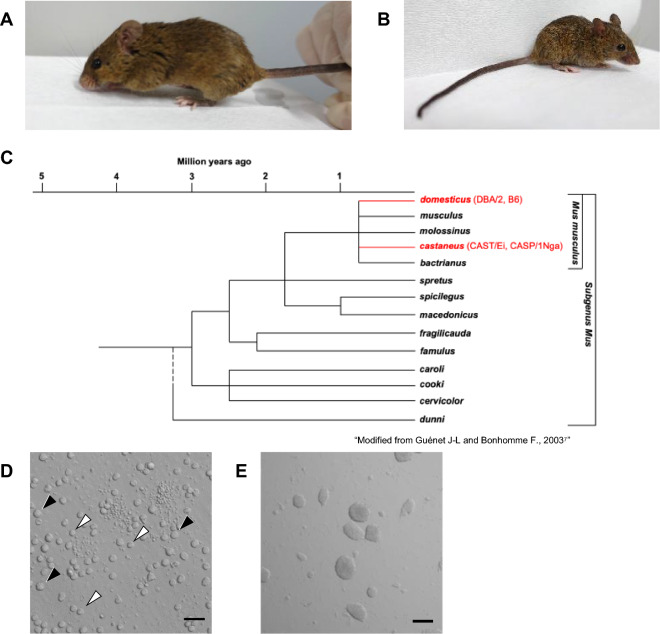
Two wild-derived mouse strains (*Mus musculus castaneus*) were used in this study. CAST/Ei (**A**) and CASP/1Nga (**B**). Both strains have agouti coat color and smaller body weight than laboratory mice. (**C**) The phylogenetic tree of mouse subspecies strains. CAST and CASP divided from laboratory mice one million years ago, but within the same *M. musculus* species as *domesticus* (DBA/2 and B6). (**D**) Donor cell nuclei collected from caudal vein peripheral blood. Black arrowheads indicate leukocytes (putative granulocytes and monocytes) used as nuclear donors. White arrowhead: lymphocytes that have smaller cell sizes than granulocytes and monocytes. Bar = 20 µm. (**E**) ntESCs established from leukocytes SCNT embryos. Bar = 200 µm.

As a preliminary experiment, we first compared SCNT embryos derived from cumulus cells and peripheral blood cells for the efficiencies of preimplantation stage development and ntESC establishment using laboratory mice. We collected these donor cells from B6D2F1 mice, the most commonly used laboratory mouse strain for SCNT nuclear donors. The cleavage rates of leukocyte-derived embryos were 52% and 41% in males and females, respectively, being significantly lower than those of SCNT female embryos from cumulus cells mainly because of cytoplasmic fragmentation of reconstructed oocytes shortly after activation (85%, Table 1). This was most likely due to the frequent failure of nuclear membrane breakage of leukocytes at nuclear transfer, consistent with our previous study^18,19^. The developmental rate into blastocysts of leukocyte-derived embryos was 26% (13/50) and 40% (23/58) per cleaved embryo in males and females, respectively, which was comparable to that of cumulus-derived embryos (41/127, 32%). When these blastocysts were transferred into ESC culture medium containing 15% KSR and 2i/LIF, 8/13 (62%) and 14/23 (61%) of them successfully developed into ntESC lines in males and females, respectively. We could also establish ntESC lines from cumulus-derived blastocysts (6/17, 35%). From these results, we confirmed that leukocytes could be used for ntESC establishment as reported for cumulus cells. Next, we attempted to use peripheral leukocytes from wild-derived strains, CAST/Ei (CAST, Fig. 1A) and CASP/1Nga (CASP, Fig. 1B), as nuclear donors. They belong to *M. m. castaneus*, a subspecies of laboratory mice (*M. m. domesticus*) (see Fig. 1C for the genealogical tree) and their maintenance is relatively inefficient due to their low reproductive performance even with ART^10^. Donor leukocytes of these wild-derived mice could be readily isolated by treating the whole blood cells with RBC-lysing buffer (Fig. 1D, see “Methods”). After nuclear transfer, 50–64% of reconstructed embryos developed into 2-cells, irrespective of sex and strain (Table 1). Of them, 17% and 8% in CAST and 31% and 18% in CASP male and female embryos, respectively, reached blastocysts, which were comparable with those of B6D2F1 donor experiments (Table 1). Finally, we could obtain 11 (8 male and 3 female) and 13 (5 male and 8 female) ntESC lines from CAST and CASP mice, respectively (Table 1, Fig. 1E).

**Table 1:** Developmental efficiencies of SCNT embryos and ntESC derivation.

Strain (*Species name*)	Donor cell	Sex	No. embryos cultured	No. embryos at 24 h (%/No. cultured)		No. blastocysts (%/No. 2-cell)	No. established ntESC lines (%/No. blastocysts)
				Fragmentation	2-cell		
B6D2F1 (*M. m. domesticus*)	Cumulus	F	150	18 (12)^a,b^	127 (85)^a^	41 (32)^a^	6/17 (35)^b^
	Leukocyte	M	96	30 (31)^a′^	50 (52)^a′^	13 (26)	8 (62)
	Leukocyte	F	140	64 (46)^a′,c^	58 (41)^a′,c,d^	23 (40)^c,d^	14 (61)
CAST/Ei (*M. m. castaneus*)	Leukocyte	M	166	59 (36)^b′^	83 (50)^a′^	14 (17)^a′^	8 (57)
	Leukocyte	F	88	33 (38)^b′^	51 (58)^d′^	4 (8)^a′,c′^	3 (75)
CASP/1Nga (*M. m. castaneus*)	Leukocyte	M	68	18 (26)	42 (62)	13 (31)	5 (38)
	Leukocyte	F	88	24 (27)^c′^	56 (64)^c′^	10 (18)^b′,d′^	8 (80)^b′^

a, a′ p < 0.01; b, b′ p < 0.05 as compared with B6D2F1 cumulus clone.

c, c′ p < 0.01; d, d′ p < 0.05 as compared with same sex.

### Gene expression patterns and the chromosomal integrity in the inter-subspecies ntESC lines

**Figure 2:**
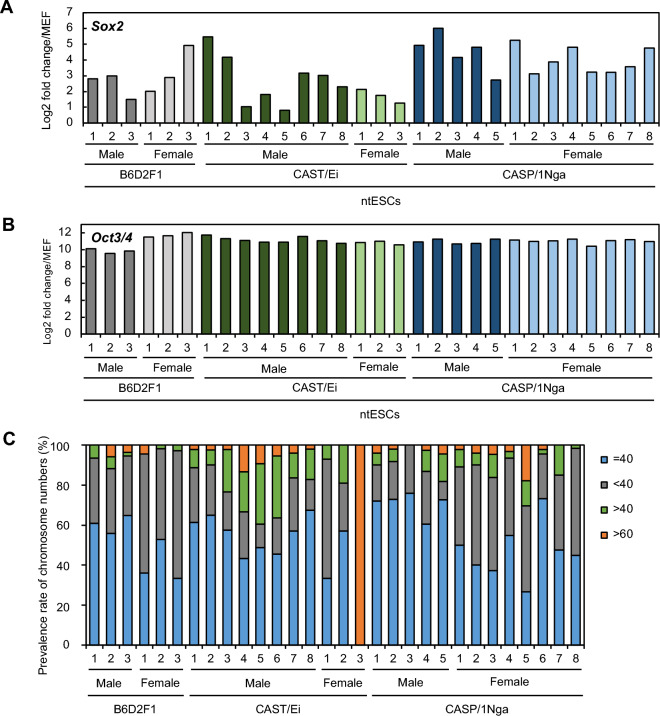
(**A**,**B**) Relative gene expression levels of pluripotent marker genes, *Sox2* (**A**) and *Oct3/4* (**B**). Gene expression levels were normalized with *Canx*. (**C**) Chromosome numbers of established ntESC lines. 2n = 40 (light blue) is the normal chromosome number.

In the next series of experiments, we determined characteristics of the ntESC lines established from leukocytes from two wild-derived subspecies strains. All ntESC lines showed typical ESC colonies with a compacted, dome-shape morphology (Fig. 1E). They expressed mouse pluripotency markers, *Sox2* and *Oct3/4*, as determined by qRT-PCR (Fig. 2A,B) and both genes were more enhanced in all ntESC lines than control mouse embryonic fibroblasts (MEF). Pluripotency gene expression was also confirmed by RNA-seq analysis (below).

The proportion of cells with the normal karyotype (n = 40) in the control B6D2F1 leukocyte-derived ntESC lines was 56–65% and 33–53% in males and females, respectively (Fig. 2C). In male CAST ntESC lines, the range of the normal karyotype was 43–67%, while that in male CASP ntESC lines was 61–76%, which was higher than that of the control male BDF1 lines (Fig. 2C). In female lines, the proportion cells with the normal karyotype was more variable, ranging from 1 to 57% in CAST and from 27 to 73% in CASP, because of the presence of low-quality lines such as one line having more than 99 chromosomes in single cells (Fig. 2C). This is consistent with the fact that the number of chromosomes was generally less stable in female mouse ESC lines than in the male mouse ESC lines^20^.

**Figure 3:**
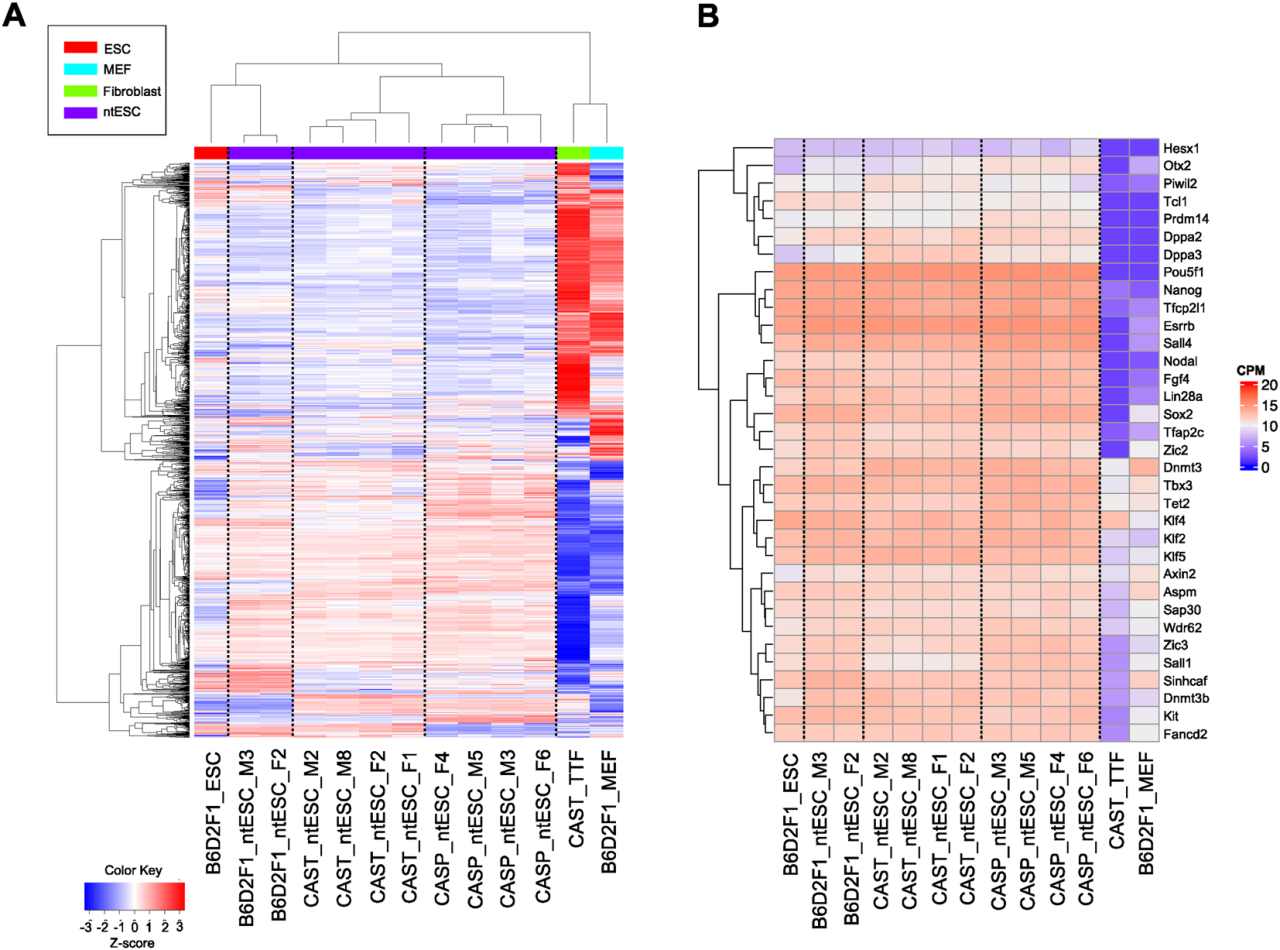
Hierarchical clustering heatmap based on RNA-seq analysis. (**A**) Heatmap showing the expression levels of top 2000 genes. The image was generated by R (v4.2.1) (**B**) Heatmap showing the expression levels of stem cell marker genes. The image was generated by ComplexHeatmap (v2.13.1, https://jokergoo.github.io/ComplexHeatmap-reference/book/).

We next performed genome-wide gene expression analysis by RNA sequencing using two male (#M2 and #M8 in CAST, #M3 and #M5 in CASP) and two female (#F1 and #F2 in CAST, #F4 and #F6 in CASP) ntESC lines with the normal karyotype from each strain. One male and one female leukocyte-derived ntESC line from B6D2F1 mice were used as controls. A hierarchical clustering analysis revealed that all the ntESC lines were primarily separated from tail-tip and embryonic fibroblast cells and were secondarily separated from each other based on the strains (Fig. 3A). However, the expression patterns of pluripotency marker genes of ntESC lines of CAST and CASP were indistinguishable from those of B6D2F1 ntESCs or fertilization-derived ESCs, indicating that they were of the same quality in terms of pluripotency gene expression (Fig. 3B). The expression patterns of differentiation marker genes were repressed or weakly expressed in all ntESC lines and a fertilization-derived ESC line (Supplementary Fig. S1).

### Determination of the genetic origin and the mitochondrial DNA origin in ntESC lines

**Figure 4:**
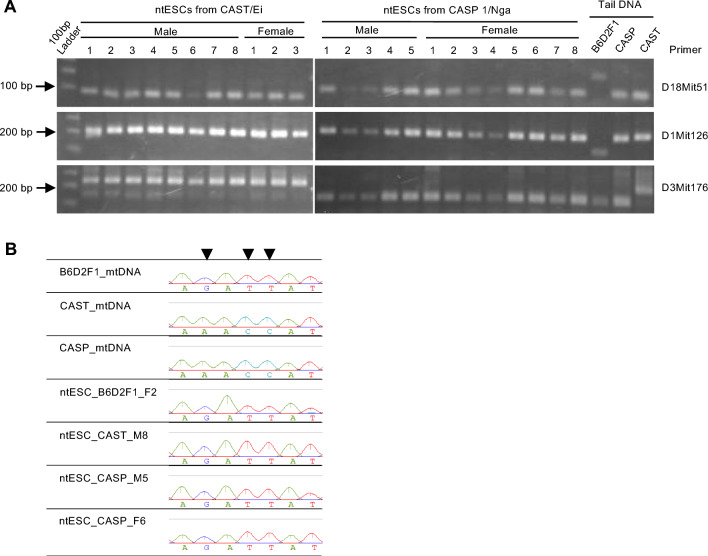
Analysis of the origin of genomic DNA and mitochondrial DNA (mtDNA) in ntESCs established in this study. (**A**) Microsatellite analysis for the genomic DNA of all established ntESC lines. D18Mit51 and D1Mit126 discriminate between laboratory mice (B6D2F1) and wild-derived mice (CASP and CAST). D3Mit176 discriminates between CASP and CAST. (**B**) Representative results of mtDNA sequence analysis of four ntESC lines. Primer sequences used are shown in Supplementary Table S1.

To confirm that the leukocyte nuclei were correctly transferred into enucleated oocytes, we examined the genetic origin of all ntESC lines by simple sequence length polymorphisms (SSLPs). We used two types of primer sets, (D18Mit51 and D1Mit126), which could differentiate between the B6D2F1 (*M. m. domesticus*) and CAST/CASP (*M. m. castaneus*) genotypes. The results confirmed that all ntESC lines generated were derived from nuclei of *M. m. castaneus* cells (Fig. 4A, Supplementary Figs. S2 and S3). The third primer set (D3Mit176) that could differentiate between the CAST and CASP genotypes also showed the expected genotype patterns for each strain.

Unlike the nuclear genomic inheritance from the donor cells, it is plausible that the cytoplasmic genome (mitochondrial DNA, mtDNA) could be carried over to the ntESC, as has been reported for inter-subspecies SCNT in mice^21^ and ntESCs in humans^22^ and primates^23^. Our mtDNA haplotype analysis based on the D-loop region sequence detected only the laboratory mouse (*M. m. domesticus*) patterns, but not the *M. m. castaneus* patterns in all the ntESCs, indicating the donor mtDNAs were completely replaced with the oocyte-derived mtDNAs (Fig. 4B).

### Assessment of the differentiation ability in vivo by teratoma formation assay

**Figure 5:**
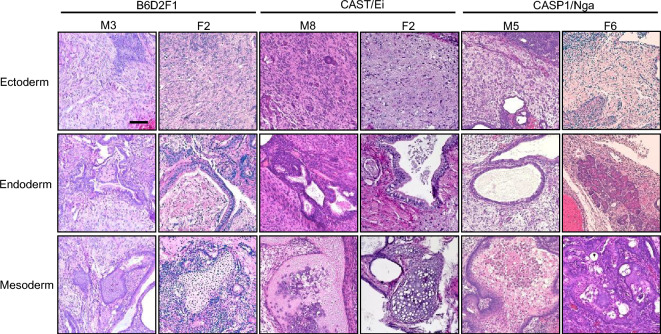
Teratoma formation assay of selected ntESC lines from each mouse strain. Differentiation into ectoderm (neural cell), endoderm (gland), and mesoderm (cartilage) was confirmed within teratoma tissues. Bar = 100 µm.

We assessed the differentiation ability in vivo of ntESC lines by teratoma formation assay. We selected ntESC lines, one each from CAST, CASP, and B6D2F1 strains of both sexes; #M8 male and #F2 female from CAST, #M5 male and #F6 female from CASP, and #M3 male and #F2 female from B6D2F1. Their single cell suspensions were injected into the hind leg of immune-deficient C.B-17/Icr-*scid/scid* mice. The time for teratoma masses to reach 10–15 mm in size was 22–56 days and the growth rate of teratoma tended to be faster in male lines than in female lines (data not shown). All teratoma masses from six ntESC lines showed extensive differentiation into all three embryonic germ layers (Fig. 5), including neural cells (ectoderm), glands (endoderm), and cartilage (mesoderm).

### The germline transmission competence of inter-subspecies ntESCs

**Figure 6:**
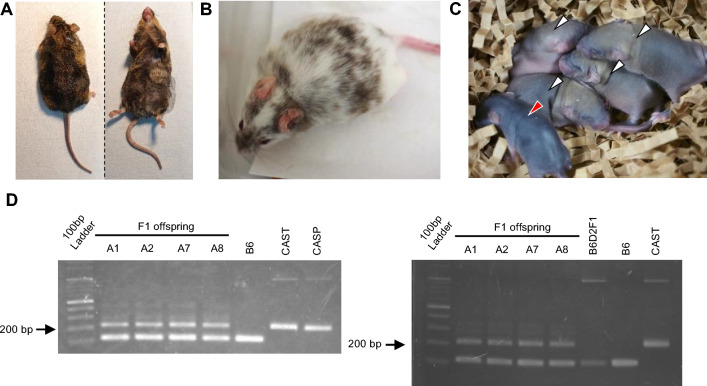
Chimeric mice and their offspring. (**A**) Appearance of chimeric mice (#12) derived from a CAST ntESC line (#M8) and (B6D2F1 × B6) F1 recipient embryos, which produced agouti and black coat colors, respectively. (**B**) Appearance of a chimeric mouse (#36) derived from a CAST ntESC line (#F2) and an (ICR × ICR) recipient embryos, which produced agouti and white (albino) coat colors, respectively. (**C**) Five F1 offspring between a B6 female and a CAST chimeric male (#23). Four pups with agouti coat color (#A3 to #A6, white arrowheads) carried the genome from the CAST ntESC line (#M8). The remaining black pup carried the host embryo genome ([B6D2F1 × B6]F1) (red arrowhead). (**D**) Microsatellite analysis of four F1 offspring (#A1, 2, 7, 8) generated by mating of a CAST chimeric male (#12 and #23) and B6 female, showing that the ntESC genome (#M8) was inherited to the F1 offspring. Microsatellite markers used are D1Mit126 (left) and D3Mit176 (right).

Finally, the germline transmission competence of ntESC lines was examined by generation of chimeric offspring. We used the same ntESC lines as the teratoma forming assay (above). In the first series of experiments, we transferred ntESCs of both sexes from two strains into host blastocysts with ICR or (B6D2F1 × B6)F1 genetic background. Each experimental group consisted of 18–66 embryos transferred into uteri of recipient females. Chimeric mice were identified by their coat color, i.e., the mixture of agouti color (CAST and CASP) from ntESCs and albino (ICR) or black ([B6D2F1 × B6]F1) color from host embryos (Supplementary Fig. S4). From the male CAST line, a total of 14 chimeric mice were obtained from 34 mice born, irrespective of the host embryo genotype (Fig. 6A**, **Table 2). From the female CAST line, 2 chimeric mice were obtained among 32 newborns (Fig. 6B**, **Table 2). Similarly, 4 chimeric mice were born from the male CASP line, but none was obtained from the female CASP lines (Table 2). We also tested whether 8-cell embryos could be better recipients for some ntESC lines, but only 1 chimeric male was born from female CAST ntESC line (#F2) (Table 2). The chimeric contribution of ntESCs greatly varied with the chimeric individual mice, ranging from a few percent to 60% based on the hair color pattern. Furthermore, it is known that functional ESC-derived gametes can be produced in chimeric mice only when the ESCs and host embryos are the same sex^24^. Therefore, for the germline transmission experiments, we used ntESC-derived chimeric mice showing > 30% chimerism and the external appearance sex-matched with the ntESC line. The chimeric mice thus selected were mated with mice of the opposite sex from the strain of host embryos, so that the germline transmission of the ntESC genome could be confirmed by the agouti coat color of the F1 offspring (Supplementary Fig. S4, Supplementary Table S2). As a result, we obtained ntESC-derived offspring with agouti coat color from two male CAST chimeric mice (#12 and #23) derived from a male CAST ntESC line (#M8), indicating that at least one male CAST ntESC line (#M8) had germline transmission competence (Fig. 6C). The ntESC origin of these F1 offspring was further confirmed by SSLPs that showed the presence of CAST and laboratory mouse alleles, originating from father and mother, respectively (Fig. 6D, Supplementary Fig. S5). Thus, we succeeded in restoring the wild-derived CAST genome in offspring using a ntESC line produced from a peripheral leukocyte in a living male CAST mouse.

**Table 2:** Production of chimeric mice from ntESC lines.

Host embryo (female × male)	ntESCs line		Stage of recipient embryo	No. embryos transferred (ET)	No implantation (%/No. ET)	No fetuses (%/No. ET)	No. chimeric mice		
							Mice (%/No. fetuses)	Male	Female
ICR × ICR	CAST/Ei	M8	Blastocyst	53	24 (45)	11 (21)	5 (46)	4	1
		F2	Blastocyst	54	36 (67)	24 (44)	2 (8)	0	2
			8-cell	58	31 (53)	6 (10)	1 (17)	1	0
	CASP/1Nga	M5	Blastocyst	59	51 (86)	25 (42)	2 (8)	2	0
			8-cell	43	29 (67)	3 (7)	0 (0)	0	0
		F6	Blastocyst	50	40 (80)	19 (38)	0 (0)	0	0
			8-cell	40	35 (88)	16 (40)	0 (0)	0	0
B6D2F1 × B6	CAST/Ei	M8	Blastocyst	54	49 (91)	23 (43)	9 (39)	5	4
		F2	Blastocyst	25	18 (72)	8 (32)	0 (0)	0	0
			8-cell	60	34 (57)	13 (22)	0 (0)	0	0
	CASP/1Nga	M5	Blastocyst	66	41 (62)	13 (20)	2 (15)	2^a^	0
			8-cell	38	33 (87)	20 (53)	0 (0)	0	0
		F6	Blastocyst	18	10 (56)	5 (28)	0 (0)	0	0
			8-cell	36	12 (33)	8 (22)	0 (0)	0	0

^a^These mice were cannibalized during lactation.

## Discussion

In this study, we first demonstrated that ntESCs from wild-derived mouse strains could be successfully established and that their genome could be inherited into the next generation through germline transmission of chimeric mice. To derive germline-competent ntESCs from wild-derived mice, we needed to overcome at least three technical hurdles. The first was the selection of appropriate donor cell types for SCNT. Mouse cloning is usually performed using cumulus cells or immature Sertoli cells, which are collected from euthanatized animals. Such donor cells are not appropriate for our purpose of conserving the valuable genetic resources of wild-derived mice. Therefore, in this study, we chose leukocytes (granulocytes or monocytes) for nuclear donors of ntESCs because they can be collected from a drop of blood from the tail tip of live mice^18^. Generally, leukocytes are difficult to clone by SCNT, except for natural killer T cells^25–27^. However, it is known that at least some of the leukocyte-derived SCNT embryos can develop into blastocysts. Indeed, B lymphocytes and T lymphocytes could be used to establish ntESCs for the purpose of generation of “clonal” mice via chimeric mouse production^28^. In this study, we also successfully obtained enough numbers of SCNT blastocysts from peripheral leukocytes to generate ntESCs. Unlike the previous clonal mouse study, it is now very common to treat SCNT embryos with trichostatin A (TSA), a potent histone deacetylate inhibitor, to enhance their developmental ability^29,30^. Therefore, we may expect that blastocysts will also be obtained from other wild-derived strains following SCNT using peripheral leukocytes.

The second technical hurdle was the derivation of ntESCs from inter-subspecies SCNT blastocysts. It is generally known that inter-(sub)species SCNT is sometimes technically challenging^31^ and is successful in only a limited number of animal species. As far as we know, there are no successful reports of inter-(sub)species SCNT in rodents. Therefore, it was possible that blastocysts produced by inter-subspecies SCNT from wild-derived strains would be of low quality for ntESC derivation. Furthermore, we did not know if the culture conditions for derivation of ESCs in laboratory mice could also be applied to the wild-derived strains. In this study, we found that the inter-subspecies SCNT blastocysts had sufficient quality and ntESC could be generated under the same conditions as laboratory mice using LIF/KSR/2i. We obtained 3–8 lines from SCNT blastocysts (38–80% derivation rates) in four experimental groups (2 strains × 2 sexes) (Table 1). These data suggest that the LIF/KSR/2i system was also effective for derivation of ntESCs from wild-derived strains (*M. m. castaneus*), although there was some experiment-to-experiment variation.

Third, restoration of wild-derived mice from ESCs through production of chimeric mice is also an inter-subspecies system because we prepared host embryos and recipient females from laboratory mice; it is impractical to use wild-derived mice for these purposes. According to Araki et al., ESCs from the wild-derived MSM/Ms strain (*M. m. molossinus*) better contributed to chimeric mice following injection into ICR blastocysts than aggregation with ICR morulae^14^. The authors also showed that use of blastocysts of (C57BL/6 × B6D2F1)F1, instead of ICR blastocysts, slightly improved the chimera efficiency. Based on these results, we injected CASP or CAST ntESCs into blastocysts from ICR or (B6D2F1 × B6)F1 and obtained chimeric mice from male lines from both strains (Table 2). We also found that use of pseudopregnant ICR females as recipients did not compromise development of chimeric embryos. We have previously reported that transfer of embryos from the MSM strain and SPR2 (*M. spretus*) into laboratory females needed cyclosporin A, an immunosuppressant, or (B6 × C3H) recipient females to avoid immuno-rejection by mothers^32,33^. It was probable that the origin of the placenta from laboratory mice in our chimeric mice could minimize the immunological attack from the mother. Consequently, we confirmed at least one male CAST ntESC line was competent for germline transmission by mating with B6 females.

The female ntESC lines established in this study were mostly of lower quality than their male counterparts in terms of the normal karyotype, the period necessary for teratoma formation, and the germline transmission ability. This sex-dependent difference may be common to ESCs from laboratory mice. It is known that male ESCs have higher developmental potential than female ESCs, more likely producing offspring through germline transmission^20^. Furthermore, most of the mouse ESCs established by conventional LIF/serum conditions were males^34^. Thus, male ESCs are superior to female ESCs in the establishment efficiency as well as their quality as pluripotent stem cells, but the underlying mechanisms remain to be understood. Hypomethylated DNA at the differentially methylated regions was responsible for the poor quality of female ESCs^20^, but other factors might also be involved. Thus, it would be difficult to establish germline-competent female ntESCs from wild-derived mice through currently available technologies. Another way to generate such ntESCs would be selective proliferation of XO populations from the existing male XY ntESCs. It is known that XO females in mice are fertile, unlike XO females in humans^35^. If we can proliferate XO female cells clonally to establish high-quality female ntESC lines, we will be able to restore wild-derived strains by natural mating of chimeric mice of both sexes. Use of germline-deficient gene-edited blastocysts using multiple guide RNAs as host embryos may more reliably yield offspring of wild-derived mice^36^.

Successful cloning by inter-species SCNT has been achieved in many families of animals, including cattle, sheep, dogs, cats, and camels. In these cases, birth rates ranged from 1 to 14%^37^. To the best of our knowledge, only two studies have demonstrated the success of inter-subspecies cloning in wild animals. In one study, two wolves (*Canis lupus*) were obtained from 251 reconstructed embryos using domestic dog oocytes (*Canis lupus familiaris*), resulting in a 1.1% birth rate^38^. Similarly, 17 African wild cats (*Felis silvestris lybica*) were produced from 1552 reconstructed embryos using domestic cat oocytes (*Felis silvestris catus*), also achieving a 1.1% birth rate^39^. Moreover, the birth rates of intra-species cloning in domestic dogs^40^ and cats^41^ were 0.2% (two puppies from 1095 reconstructed embryos) and 1.1% (one kitten per 87 reconstructed embryos), respectively. Thus, the efficiencies of inter-subspecies cloning were comparable to those of intra-species cloning. In the case of cloning wild-derived mice by inter-subspecies SCNT, the production of cloned individuals is highly challenging because they have an inbred genetic background that strongly hampers postimplantation embryonic development^25^. The developmental ability of blastocysts following SCNT of laboratory inbred strains ranges from 25 to 40% for cumulus cell cloning and 11% for tail-tip fibroblast cloning^42^, which are both comparable to our results with inter-subspecific wild-derived mice (Table 1). Based on these findings, it can be concluded that the cloning efficiencies between inter-subspecies and intra-species are not significantly different.

In conclusion, we first demonstrated that *M. m. castaneus* mouse ntESCs could be established from terminally differentiated leukocytes by inter-subspecies SCNT. All ntESC lines we investigated expressed pluripotency markers and contributed to three germ layers. At least one male ntESC line was proven to be competent to transmit the genome to the next generation through the germline. Generation of ntESC from wild-derived mice could provide an alternative strategy for the backup of these invaluable mouse strains.

## Methods

### Animals

B6 (C57BL/6NCrSlc) and B6D2F1 ([C57BL/6NCrSlc × DBA/2CrSlc]F1) mice were purchased from Japan SLC. ICR mice were purchased from CLEA Japan Inc. Wild-derived mouse strains, CASP/1Nga (*M. m. castaneus*, RBRC03108, Fig. 1A) and CAST/Ei (*M. m. castaneus*, RBRC00733, Fig. 1B) were provided by RIKEN BRC. Female C.B-17/Icr-scid/scidJcl mice (SCID mice, CLEA Japan Inc., Tokyo, Japan) were used as the hosts for transplantation in the teratoma formation assay. Animals were provided with water and commercial laboratory mouse chow ad libitum and were housed under a controlled lighting condition (daily light from 07:00 to 21:00). For euthanasia, cervical dislocation was performed by trained individuals. Prior to the operation, mice were deeply anesthetized by intraperitoneal injection of tribromoethyl alcohol (avertin) or a mixture of three types of anesthetic agents (medetomidine, midazolam and butorphanol). They were maintained under specific pathogen-free conditions. The care and use of animals in this study were performed according to the guidelines for the use and maintenance of experimental animals from the Japanese Ministry of Environment. All animal experiments included in this study were approved by the Institutional Animal Care and Use Committee of RIKEN Tsukuba Branch. This study is reported in accordance with ARRIVE guidelines (https://arriveguidelines.org).

### Nuclear transfer

Nuclear transfer was performed following a previous report with slight modification^43^. For collecting oocytes, eight-week-old or older B6D2F1 females were injected with 7.5 IU of equine chorionic gonadotropin (eCG, Nippon Zenyaku Kogyo Co., Ltd., Fukushima, Japan) and 7.5 IU human chorionic gonadotropin (hCG, ASKA Pharmaceutical Co., Ltd., Tokyo, Japan) at an interval of 48 h. Fifteen hours later, cumulus-oocyte complexes were collected and treated with KSOM medium containing 0.1% bovine testicular hyaluronidase to remove cumulus cells. Oocytes were enucleated in Hepes-KSOM medium containing 7.5 µg/ml cytochalasin B (250233, Merck, Darmstadt, Germany) by a piezo-driven micromanipulator (PMAS-CT150, PRIMTECH Corp., Ibaraki, Japan). Donor peripheral leukocytes were prepared as previously reported^18^. Briefly, about 50 µL of peripheral blood was collected from the tail vein, and red blood cells (RBC) were lysed with RBC lysis buffer (155 mM NH_4_Cl, 10 mM KHCO_3_, 2 mM EDTA, pH 7.4). For nuclear transfer, leukocytes with diameter > 8 µm were selected as monocytes or granulocytes and injected into enucleated oocytes by a piezo-driven micromanipulator. One hour after injection, nuclear transferred embryos were activated with Ca^2+^-free KSOM containing 2.5 mM SrCl_2_, 50 nM trichostatin A (TSA, T8552-1MG, Merck), and 5 μM latrunculin A (LatA, L5163, Merck). They were further cultured in KSOM containing TSA and LatA for seven hours. After washing, embryos were cultured in KSOM at 37 °C under 5% CO_2_ in air until the blastocyst stage.

### Establishment and maintenance of ntESCs

Establishment and maintenance of ntESCs were performed as described previously with some modifications^44^. NtESC were established on feeder cells. MEF (mouse embryonic fibroblast) from C57BL/6NCrSlc were cryopreserved after mitomycin C (M0503-2MG, Merck) treatment and used as feeder cells. The zona pellucida of SCNT blastocysts was removed by acid Tyrode’s solution (T1788, Merck). The zona-free blastocysts were cultured with KnockOut-DMEM including 15% Knockout Serum Replacement (10828028, Thermo Fisher Scientific K.K., Massachusetts, USA), 1 × Non-Essential Amino Acid (11140050, Thermo Fisher Scientific K.K.), 1 × GlutaMAX supplement (32571036, Thermo Fisher Scientific K.K.), 0.1 mM 2-Mercaptoethanol (M-7522, Merck), 10^3^ U/mL Leukemia Inhibitory Factor (LIF, ESG1107, Merck), 3 mM CHIR99021 (04-0004, Reprocell Inc., Kanagawa, Japan) and 1 µM PD0325901 (04-0006, Reprocell Inc.) on feeder cells in a 37 °C 5% CO_2_ incubator. The fully grown colonies were passaged and cultured under the same conditions until the experiments. All ntESC lines were subjected to analysis within 15 passages.

### Chromosomal number analysis

The ntESCs established were seeded into a 6-well tissue culture dish and cultured overnight. They were incubated with Karyo MAX COLCEMID (15210-040, Thermo Fisher Scientific K.K.) in culture medium for five hours. Cells dispersed by 0.25% trypsin treatment were collected by centrifugation, mixed with 3 mL of 0.075 M KCl, and then warmed for 20 min in a 37 °C water bath. For fixation, a double volume of Carnoy’s solution (methanol: acetic acid = 3:1) was added, then incubated at 4 °C overnight. Cells were collected by centrifugation and washed with Carnoy’s solution two times. Cells suspended in Carnoy’s solution were dripped onto glass slides from a height of 50 cm. After drying, the slides they were stained with Giemsa stain solution (1.09204.0130, Merck) for 10 min and washed with water.

### Quantitative reverse transcription PCR (qRT-PCR)

NtESCs were collected and left in gelatin-coated culture dish for 1 h twice to remove feeder cells. Total RNA was extracted by RNeasy Mini Kit (74104, QIAGEN, Hilden, Germany). Then, cDNA was synthesized from total RNA by SuperScript IV First-Strand cDNA Synthesis Reaction (18090050, Thermo Fisher Scientific K.K.). Real time (quantitative) PCR was performed by mixing cDNA, PowerUP SYBR Green Master Mix (A25776, Thermo Fisher Scientific K.K.), and primers. The primers information was shown in Supplementary Table S1.

### Simple sequence length polymorphisms (SSLP) analysis

NtESCs were collected and left in gelatin-coated culture dish for 1 h twice to remove feeder cells. DNA samples were extracted from mice tails using Wizard Genomic DNA Purification Kit (A1125, Promega Corp., Wisconsin, USA) according to the manufacturer’s instructions. We searched microsatellites of each strain using the Mouse Microsatellite Data Base of Japan (MMDBJ) and selected three sites with the largest fragment differences in their size (Supplementary Table S1). Microsatellite primers were prepared following Mouse Genome Informatics. PCR products were amplified by Tks Gflex DNA Polymerase (R060B, Takara Bio Inc., Shiga, Japan) and compared by electrophoresis.

### RNA sequencing and analysis

NtESCs were collected and left in gelatin-coated culture dish for 1 h twice to remove feeder cells. Total RNA was extracted in the same method described above. The library was prepared with TruSeq stranded mRNA Library Prep Kit (Illumina, San Diego, USA). Sequencing (100 base pair paired-end sequencing) was performed using the Illumina NovaSeq 6000 platform (Illumina). Low-quality reads were removed with fastp (0.22.0) for further analysis. Retained reads were aligned with the reference mouse genome (GRCm38) using HISAT2 (2.2.1) and transcript assembly and quantifications were performed with StringTie (2.1.7). All count data were normalized with DESeq2 and transformed into count per million (CPM) values.

### Teratoma formation assay

NtESCs (> 1.0 × 10^6^ cells) suspended in phosphate-buffered saline without Ca^2+^ and Mg^2+^ were injected into the hind leg muscle of SCID mice. Enlarged teratomas (10–15 mm in length) were removed and fixed with 10% formalin neutral buffer solution. After fixation, tissue samples were embedded into paraffin and sectioned into 4 µm thick slices, and then stained by Hematoxylin–Eosin solution. Images were acquired using HS All-in-one Fluorescence Microscope (BZ-X800, Keyence, Osaka, Japan).

### Generation of chimeric mice

IVF embryos (ICR × ICR or B6D2F1 × B6) at the 8-cell or blastocyst stages were used as host embryos. Three to five ntESCs were injected into 8-cell embryos and 5–10 ntESCs were injected into blastocysts by a piezo-driven micromanipulator. After injection, embryos that reached the blastocyst stage were transferred into the uteri of 2.5 dpc pseudopregnant ICR females. The offspring were retrieved by cesarean section at 19.5 dpc. Of them, mice with a high chimerism were used for mating to indicate the germline transmission competence of the original ntESC lines (for details, see “Results”).

### Statistical analysis

Embryo developmental rates were analyzed by chi-square test. *P* values < 0.05 were considered statistically significant.

## Supplementary Information

Supplementary Information.

## Data Availability

The data underlying this article are available in GSE225058, at Gene Expression Omnibus (https://www.ncbi.nlm.nih.gov/gds/).
