# Supplementary material for: Derivation of embryonic stem cells from wild-derived mouse strains by nuclear transfer using peripheral blood cells
Source: Sci Rep. 2023 Jul 10;13:11175. doi: 10.1038/s41598-023-38341-0 (PMC10333218; doi:10.1038/s41598-023-38341-0)
Supplement: Supplementary file 1 — Supplementary Information. [file 41598_2023_38341_MOESM1_ESM.pdf]

Supplementary Information

Derivation of embryonic stem cells from wild-derived mouse strains by nuclear transfer using peripheral blood cells

Naomi Watanabe[1,2], Michiko Hirose[1], Ayumi Hasegawa[1], Keiji Mochida[1], Atsuo Ogura[1,2], Kimiko Inoue[1,2]

[1]RIKEN BioResource Research Center, Tsukuba, Ibaraki, Japan, [2]Graduate School of Science and Technology, University of Tsukuba, Tsukuba, Ibaraki, Japan

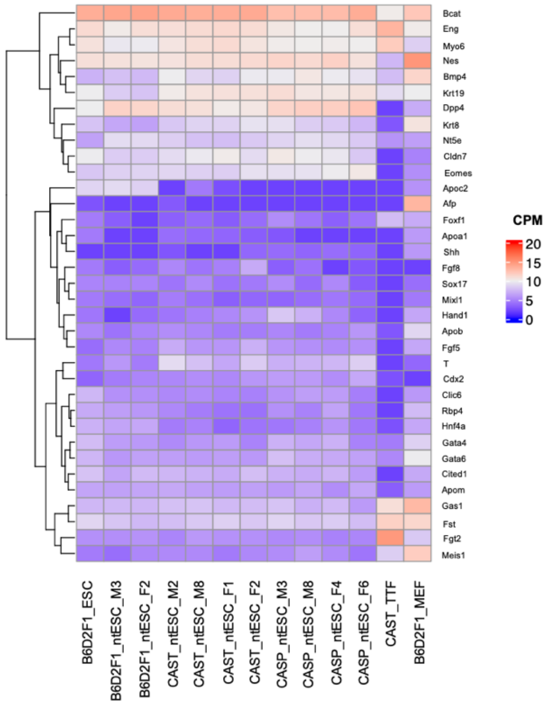

Supplementary Figure S1. Hierarchical clustering heatmap differentiation marker genes based on RNA-seq analysis.

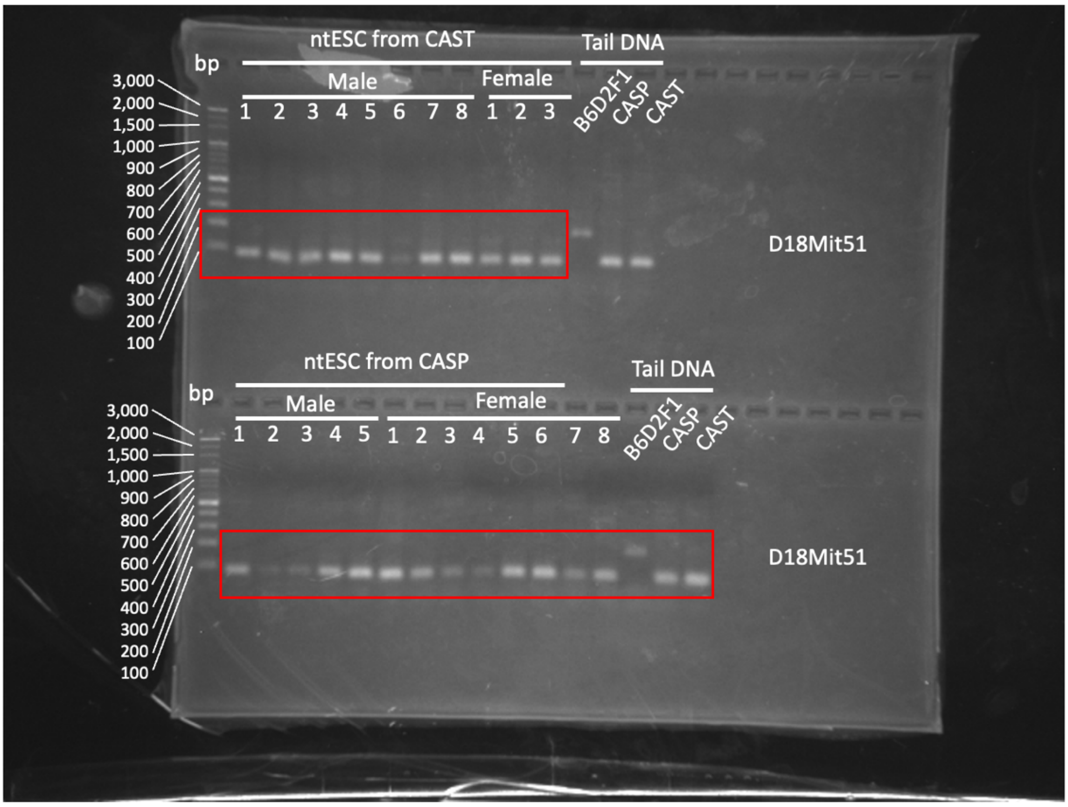

Supplementary Figure S2. The uncropped image of the upper panels of Figure 4A, which indicate the results of D18Mit51 primer set. The red frames indicate the cropped areas.

A

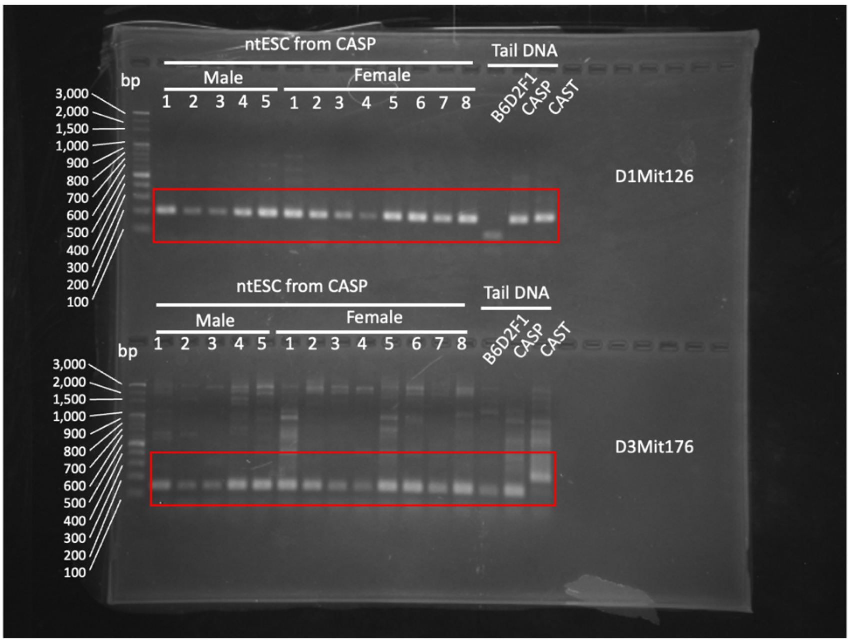

B

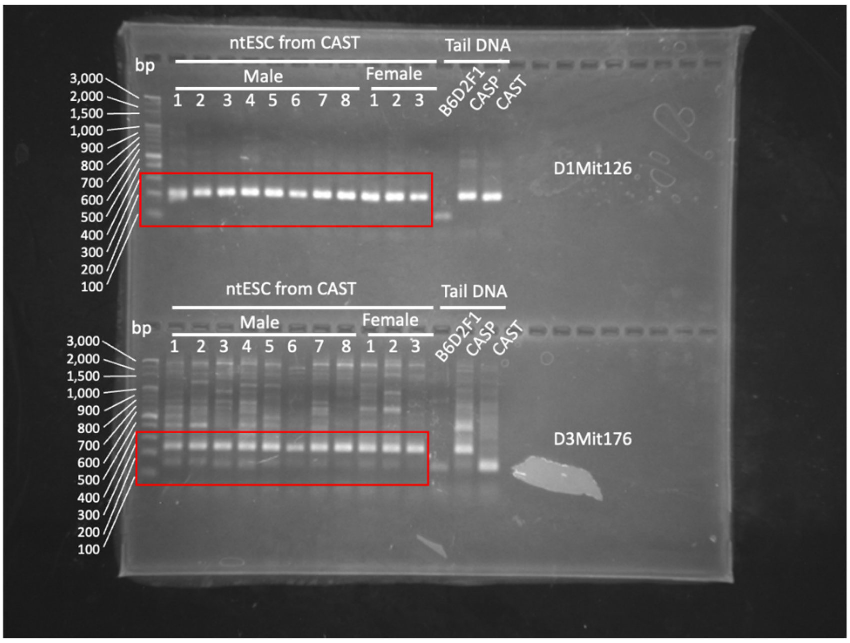

Supplementary Figure S3. (A) The uncropped image of the middle left and lower left panels of Figure 4A, which indicate the results of D1Mit126 and D3Mit176 primer sets. (B) The uncropped image of the middle right and lower right panels of Figure 4A, which indicate the results of D1Mit126 and D3Mit176 primer sets. The red frames indicate the cropped areas.

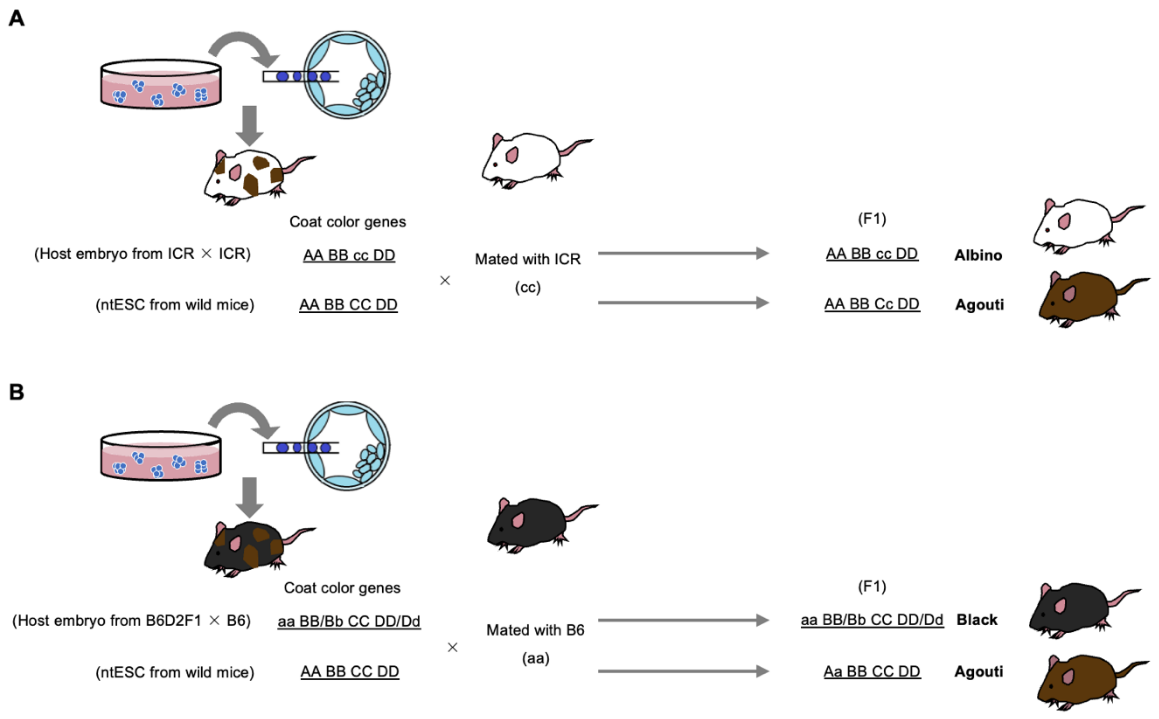

Supplementary Figure S4. Determination of germline transmission competence of ntESCs by F1 offspring coat colors. (A) The case of using ICR (AA BB cc DD) as host embryos. When chimeric mice were mated with the opposite sex of ICR, F1 offspring transmitted from ntESCs is expected to be agouti coat color (AA BB Cc DD). (B) The case of using (B6D2F1 × B6)F1 (aa BB/Bb CC DD/Dd) as host embryos. When chimeric mice were mated with the opposite sex of B6, F1 offspring is expected to be agouti coat color (Aa BB CC DD).

A

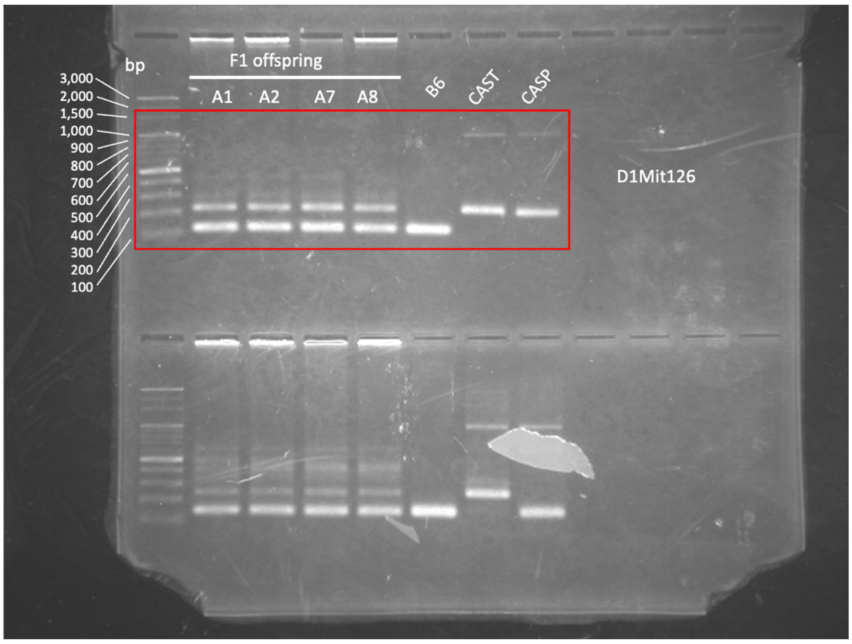

B

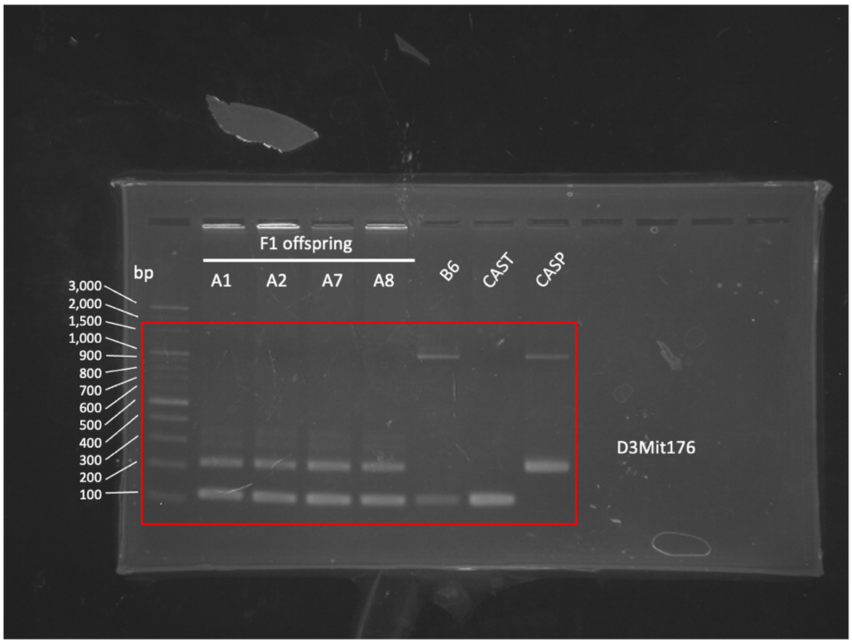

Supplementary Figure S5. (A) The uncropped image of the left panel of Figure 6D, which indicate the results of D1Mit126 primer set. (B) The uncropped image of the right panel of Figure 6D, which indicate the results of D3Mit176 primer set. The red frames indicate the cropped areas.

Supplementary Table S1. Information of primer sequences used for each analysis

experiment		Primer sequence (5' to 3')		Fragment (bp)		
		**Forward**	**Reverse**	**BDF1**	**CAST**	**CASP**
**Gene expression analysis by qPCR**	***Canx***	**TGTTGTGAACAGTGGAAATCTGC**	**AAGGGGCGTCTTCATCCCAG**			
	Oct3/4	**ATGAGGCTACAGGGACACCT**	**GACGGGAACAGAGGGAAAGG**			
	Sox2	**TAAGTACACGCTTCCCGGAG**	**ATCATGCTGTAGCTGCCGTT**			
**SSLP analysis**	**D18Mit51**	**AACATGGTGGAAACCAACTACC**	**AAGGGAAAGTCACCACATGC**	**202**	**83**	**83**
	**D1Mit126**	**GAGAGACTGGAGATATTCTTTGCC**	**CCAACCCCCCATTAAGTTCT**	**118**	**218**	**218**
	**D3Mit176**	**TTGACTCTATTCACTGGCATGC**	**CTACTCACCCAGTCTGCTTAACC**	**145**	**245**	**145**
**Determination of the mtDNA origin**		**AAAGCATCTGGCCTACACCC**	**ATGCTACCTTTGCACGGTCA**			

Supplementary Table S2. Results of the germline transmission analysis

Chimeric mice				mated with	Coat color of F1
ntESC	host embryo	sex	ID #		
CAST_M8	ICR × ICR	M	2, 3	ICR_F	Albino
CAST_M8	ICR × ICR	F	9	ICR_M	Albino
CAST_F2	ICR × ICR	M	1	ICR_F	Albino
CASP_M5	ICR × ICR	M	35	ICR_F	Albino
CASP_M5	ICR × ICR	M	36	ICR_F	No offspring
CAST_M8	B6D2F1 × B6	M	10, 11, 22	B6_F	Black
CAST_M8	B6D2F1 × B6	M	12	B6_F	Black, Agouti (A7-A10)
CAST_M8	B6D2F1 × B6	M	23	B6_F	Black, Agouti (A1-A6)
CAST_M8	B6D2F1 × B6	F	26	B6_M	No offspring
